# Comparative efficacy of two pyrethroid-piperonyl butoxide nets (Olyset Plus and PermaNet 3.0) against pyrethroid resistant malaria vectors: a non-inferiority assessment

**DOI:** 10.1186/s12936-022-04041-9

**Published:** 2022-01-11

**Authors:** Corine Ngufor, Josias Fagbohoun, Abel Agbevo, Hanafy Ismail, Joseph D. Challenger, Thomas S. Churcher, Mark Rowland

**Affiliations:** 1 London School of Hygiene and Tropical Medicine (LSHTM), London, UK; 2 Centre de Recherches Entomologiques de Cotonou (CREC), Cotonou, Benin; 3 Pan African Malaria Vector Research Consortium (PAMVERC), Cotonou, Benin; 4 Liverpool School of Tropical Medicine, Liverpool, L3 5QA UK; 5 MRC Centre for Global Infectious Disease Analysis, Infectious Disease Epidemiology, Imperial College London, Norfolk Place, London, W2 1PG UK

**Keywords:** Experimental huts, Piperonyl butoxide, PBO, Olyset plus, PermaNet 3.0, Olyset, Mixture, LLIN, Insecticide resistance, Pyrethroid-PBO, Cove, Benin, Pyrethroid resistance, Insecticide-treated nets, Long-lasting insecticidal nets, Next generation nets, Anopheles, Cove Benin

## Abstract

**Background:**

Pyrethroid-PBO nets were conditionally recommended for control of malaria transmitted by mosquitoes with oxidase-based pyrethroid-resistance based on epidemiological evidence of additional protective effect with Olyset Plus compared to a pyrethroid-only net (Olyset Net). Entomological studies can be used to assess the comparative performance of other brands of pyrethroid-PBO ITNs to Olyset Plus.

**Methods:**

An experimental hut trial was performed in Cové, Benin to compare PermaNet 3.0 (deltamethrin plus PBO on roof panel only) to Olyset Plus (permethrin plus PBO on all panels) against wild pyrethroid-resistant *Anopheles gambiae* sensu lato (s.l.) following World Health Organization (WHO) guidelines. Both nets were tested unwashed and after 20 standardized washes compared to Olyset Net. Laboratory bioassays were also performed to help explain findings in the experimental huts.

**Results:**

With unwashed nets, mosquito mortality was higher in huts with PermaNet 3.0 compared to Olyset Plus (41% vs. 28%, P < 0.001). After 20 washes, mortality declined significantly with PermaNet 3.0 (41% unwashed vs. 17% after washing P < 0.001), but not with Olyset Plus (28% unwashed vs. 24% after washing P = 0.433); Olyset Plus induced significantly higher mortality than PermaNet 3.0 and Olyset Net after 20 washes. PermaNet 3.0 showed a higher wash retention of PBO compared to Olyset Plus. A non-inferiority analysis performed with data from unwashed and washed nets together using a margin recommended by the WHO, showed that PermaNet 3.0 was non-inferior to Olyset Plus in terms of mosquito mortality (25% with Olyset Plus vs. 27% with PermaNet 3.0, OR = 1.528, 95%CI = 1.02–2.29) but not in reducing mosquito feeding (25% with Olyset Plus vs. 30% with PermaNet 3.0, OR = 1.192, 95%CI = 0.77–1.84). Both pyrethroid-PBO nets were superior to Olyset Net.

**Conclusion:**

Olyset Plus outperformed PermaNet 3.0 in terms of its ability to cause greater margins of improved mosquito mortality compared to a standard pyrethroid net, after multiple standardized washes. However, using a margin of non-inferiority defined by the WHO, PermaNet 3.0 was non-inferior to Olyset Plus in inducing mosquito mortality. Considering the low levels of mortality observed and increasing pyrethroid-resistance in West Africa, it is unclear whether either of these nets would demonstrate the same epidemiological impact observed in community trials in East Africa.

## Background

Long-lasting insecticidal nets (LLINs) remain one of the most powerful tools to reduce malaria transmission in a community and provide personal protection to the user [1, 2]. They have contributed significantly to recent reductions in malaria burden [3]. Their efficacy is however threatened by increasing resistance to pyrethroids [4, 5]; the insecticide of choice used on bed-nets owing to its safety, low cost and rapid activity on vector mosquitoes [6]. To maintain the effectiveness of insecticide treated nets for malaria control, new types of LLINs treated with alternative insecticides and compounds which can either replace or complement pyrethroids on bed-nets are urgently needed.

A new class of insecticide treated nets (ITNs) combining pyrethroids and piperonyl butoxide (PBO) (pyrethroid-PBO ITNs) have been developed [7]. PBO is a synergist that inhibits specific metabolic enzymes such as mixed-function oxidases within mosquitoes that detoxify or sequester insecticides before they can have a toxic effect on the mosquito. Pyrethroid-PBO nets can therefore induce increased mortality of pyrethroid-resistant malaria vectors that express mixed function oxidase based pyrethroid resistance mechanisms that are inhibited by the PBO in the net. These nets were given an interim endorsement as a new WHO class of vector control products in 2017 based on epidemiological data from a cluster randomized controlled trial in North Eastern Tanzania [8], that demonstrated additional malaria control with one prototype pyrethroid-PBO net (Olyset Plus) compared to a pyrethroid-only net (Olyset Net), against pyrethroid resistant malaria vectors of moderate intensity, partly conferred by monooxygenase-based resistance mechanism. Pyrethroid-PBO ITNs are conditionally recommended for malaria vector control instead of pyrethroid-only ITNs in areas of confirmed intermediate levels of resistance mediated by monooxygenase-based resistance mechanism [9]. This endorsement has been followed by an increasing uptake of pyrethroid-PBO nets worldwide [10]; in sub-Saharan Africa for example, the proportion of pyrethroid-PBO nets of all nets delivered increased from 3% in 2018 to 35% in 2021.

While Olyset Plus was the first in class pyrethroid-PBO net to demonstrate public health value as observed in the Tanzanian trial [8], there are currently five additional types of pyrethroid-PBO ITNs on the World Health Organization (WHO) list of prequalified vector control products: PermaNet 3.0, Veeralin, Tsara Boost, Tsara Plus and very recently DuraNet Plus [7]. These nets have all demonstrated superiority over pyrethroid-only nets in terms of mosquito mortality and blood-feeding inhibition in multiple experimental hut trials across Africa [11–17]. However, they differ from Olyset Plus in their design and specifications; typically, the location of PBO on the net (i.e., all panels vs. roof panel only), the type and dose of pyrethroid used, bioavailability and retention of PBO after washing and could, therefore, differ in entomological and epidemiological impact. A recent large cluster-randomized trial in Uganda, evaluating the efficacy of Olyset Plus and PermaNet 3.0 compared to pyrethroid-only nets in a setting of high pyrethroid resistance, also demonstrated better protection against malaria with pyrethroid-PBO nets compared to pyrethroid-only nets for up to 18 months confirming the findings of the Tanzanian trial [18]. While the Ugandan trial was not powered to directly compare between the different ITN brands tested, the results showed that the additional protective effect of the pyrethroid-PBO net compared to the pyrethroid-only net was initially large with PermaNet 3.0 at 6 months post-distribution but lasted only up to 12 months whereas additional protective effect with Olyset Plus appeared to have a delayed onset which was not observed at 6 months but became evident at 12 months and lasted up to 18 months. There are major differences in design between Olyset Plus and PermaNet 3.0, which could have implications on their epidemiological impact; Olyset Plus is a polyethylene net incorporated with permethrin and PBO on all panels while PermaNet 3.0 is a polyester net coated with deltamethrin with PBO restricted to the roof of the net.

To generate assurance of comparative performance of new candidate products within an established WHO vector control product class, without the need for epidemiological evidence for each new product, the WHO has developed new experimental hut study guidelines for assessing their non-inferiority to a first in class product for which evidence of public health value has already been generated [19]. These provisional guidelines are to be piloted with pyrethroid-PBO nets by comparing other WHO/PQ-listed pyrethroid-PBO nets with the first in class product, Olyset Plus. This study compared the efficacy and wash resistance of Olyset Plus and PermaNet 3.0 and assessed the non-inferiority of PermaNet 3.0 to Olyset Plus in experimental huts against wild free-flying pyrethroid resistant malaria vectors in Southern Benin.

## Methods

### Experimental hut trial

#### Experimental hut site

Experimental huts are small standardized human habitations approved by the WHO for the controlled evaluation of indoor vector control tools against wild free-flying mosquitoes. Mosquitoes enter the huts freely at night to interact with the human host and the vector control intervention and in the morning on each day of the trial, they are collected from the different compartments of each hut and scored for entomological outcomes. The experimental hut study was performed at the CREC/LSHTM experimental hut station situated in a large rice growing area in Cové, Southern Benin, where the local mosquito population has been shown to be resistant to pyrethroids [20]. The rice paddies provide extensive breeding sites for *Anopheles gambiae* throughout the year. The huts are built on concrete plinths surrounded by water-filled moats to prevent entry of scavenging ants and have veranda traps to capture the exiting mosquitoes. The walls are made of brick plastered with cement on the inside, with a corrugated iron roof. The huts have a ceiling of palm thatch and four window slits (1 cm gap) on the walls through which mosquitoes enter. The local vector population in Cove is resistant to pyrethroids and DDT and consists of a mixture of *Anopheles coluzzii* and *Anopheles gambiae *sensu stricto (s.s.), with the latter occurring at lower proportions (23%) and only in the dry season [20]. Molecular analysis revealed a L1014F kdr allele frequency of 89%. Microarray studies also found CYP6P3, a P450 validated as an efficient metabolizer of pyrethroids [21], to be overexpressed in Cove [20].

#### Insecticide resistance bioassays

To assess the frequency of pyrethroid resistance and presence of mixed function oxidases in the Cové vector population during the trial, adult mosquitoes that emerged from larvae collected from breeding sites close to experimental huts were tested in WHO cylinder bioassays with and without pre-exposure to PBO. A total of ~ 100 mosquitoes of the pyrethroid resistant *An. gambiae *s.l. Cove strain and the pyrethroid susceptible *An. gambiae* Kisumu strain were exposed to treated filter papers in WHO cylinder bioassays in batches of 25. Tests were performed with papers treated with permethrin 0.75%, alpha-cypermethrin 0.05% and deltamethrin 0.05%. To assess presence of MFO, some mosquitoes were also pre-exposed to papers treated with 4% PBO prior to exposure to insecticide-treated papers. Exposure to PBO and to insecticides lasted 1 h, knockdown was recorded after 60 min and mortality after 24 h.

#### Experimental hut treatments

Olyset Plus and PermaNet 3.0 were compared in the experimental huts when unwashed and after 20 standardized washes. A WHO-recommended pyrethroid-only long-lasting net (Olyset Net) was included to demonstrate the added effect of PBO on the insecticide-resistant local vector species. Nets were washed using savon de Marseilles and rinsed twice following WHO procedures for washing nets for experimental hut studies [22].

The following seven (7) treatments were thus tested in seven experimental huts:

1. Untreated polyethylene net
2. Olyset Net unwashed (permethrin only)
3. Olyset Net washed 20 times.
4. PermaNet 3.0 unwashed (Roof: deltamethrin plus PBO; sides: deltamethrin only)
5. PermaNet 3.0 washed 20 times.
6. Olyset Plus unwashed (permethrin plus PBO on all panels)
7. Olyset Plus washed 20 times.

#### Hut trial procedure

Treatments were allocated to the experimental huts on a weekly basis using a randomized Latin square design to adjust for any variation in hut attractiveness and minimize any carry over effect between treatments. Three replicate nets of each type were prepared, and these were rotated every 2 days on each week (6 days) of the trial. To simulate wear and tear, each net was intentionally holed with six 16 cm^2^ holes (two holes on each side and one on each end).

The trial ran for 42 nights between February and April of 2017. Consenting human volunteer sleepers slept in the huts from 9:00 p.m. to 5:00 a.m. each night and were rotated daily through the huts to account for individual attractiveness to mosquitoes. At dawn, the volunteer sleepers collected mosquitoes in the room of the hut and under the bed nets and the veranda using torches and aspirators. The mosquitoes were then transferred to the laboratory for processing where they were identified and scored for their blood feeding status, mortality and hut position. Mosquitoes were held at 27 ± 2 °C during the observations.

The following outcome measures were used to assess the efficacy of each treatment in the experimental huts:

1. Deterrence—the proportional reduction in number of mosquitoes entering huts with treated nets.
2. Exiting rates estimated from the proportions of mosquitoes collected from the verandas of treatment and control huts.
3. Mortality—the proportion of mosquitoes killed (immediate plus delayed) relative to the total collected.
4. Blood-feeding—the proportion of blood-fed mosquitoes relative to the total collected.
5. Blood-feeding inhibition—the proportional reduction in blood feeding in huts with insecticide treated nets relative to controls with untreated nets.
6. Personal protection—the proportional reduction in mosquito biting by insecticide treated nets relative to untreated nets.

### Supplementary laboratory bioassays

To help further explain the results obtained in the experimental huts, WHO cone bioassays and tunnel tests were performed on samples of netting (30 × 30 cm) obtained from Olyset Net and Olyset Plus when unwashed and after 10 and 20 washes. Washing was performed in the laboratory following WHO guidelines [22]. PermaNet 3.0 was not tested in the laboratory bioassays owing to the restricted application of PBO to the roof of the net preventing a realistic direct comparison with Olyset Plus in bioassays especially tunnel tests. Net samples from each ITN type and each wash point were tested against the following strains:

1. *An. gambiae *sensu lato (s.l.) strains from Cove, Benin (Cove strain) which is highly pyrethroid resistant. It originates from the experimental hut station in Cove and has shown > 200-fold resistance compared to the susceptible Kisumu strain in susceptibility bioassays. Resistance is mediated by elevated levels of P450s and high frequencies of *kdr* [20].
2. *An. gambiae* VKPer strain, which originated from the Kou Valley in Burkina Faso. VKPer has moderate levels of pyrethroid resistance mediated only by high frequencies of *kdr*.
3. *An. gambiae *s.s. Kisumu strain, a reference susceptible strain which originated from Kisumu Kenya.

Approximately two hundred 2–5 days old mosquitoes of each strain were exposed for 3 min in cone bioassays to four net samples of each net type in cohorts of 5 mosquitoes per cone. Knock down in cone bioassays was recorded after 1 h and mortality after 24 h.

Two to three hundred 5–8 days old mosquitoes of each strain were also exposed to each net type in tunnel tests in replicates of 50 mosquitoes per net sample. The tunnel test is a laboratory assay designed to simulate natural host-seeking behaviour of mosquitoes at night in the presence of a net. It consists of a square glass cylinder (25 cm high, 25 cm wide, 60 cm in length) divided into two sections by means of a netting frame fitted into a slot across the tunnel. An anesthetized guinea pig was housed unconstrained in a small cage in one section, and mosquitoes were released in the other section at dusk and left overnight. The net samples were holed with nine 1-cm diameter holes to allow host-seeking mosquitoes to penetrate the baited chamber; an untreated net sample served as the control. The tunnels were kept overnight in a dark room at 25–29 °C and 75–85% RH. The next morning, the numbers found alive or dead, fed, or unfed, in each section were recorded. Live mosquitoes were provided with sugar solution and delayed mortality recorded after 24 h. The guinea pigs used in this study were kept in accordance with institutional guidelines for animal care.

### Chemical analysis

At the end of the experimental hut trial, five pieces of netting (25 × 25 cm) obtained from the panels of replicate nets of each net type (before and after washing) used in the huts were assessed for deltamethrin, permethrin and PBO content using HPLC. Insecticide was extracted from each net piece with an area of 48 sq cm collected from the five net samples (25 × 25 cm) obtained from each whole net. The insecticide content of each sample was determined by injecting ten μl aliquots of the extract on a reverse-phase Hypersil GOLD C18 column (75 Å, 250 × 4.6 mm, 5-μm particle size; Thermo Scientific) at room temperature. A mobile phase of 70% acetonitrile in water was used at a flow rate of 1 ml min^−1^ to separate the target analyte. Chromatographic peaks of the insecticides and internal standard were detected at a wavelength of 232 nm with the Ultimate 3000 UV detector and analysed with Dionex Chromeleon™ 6.8 Chromatography Data System software. Quantities of insecticide were calculated from standard curves established by known concentrations of the insecticide authenticated standards and corrected by internal standard readings in each sample relative to control.

Data from chemical analysis was used to calculate the percentage retention of each active ingredient after 20 washes relative to the unwashed net and the wash retention index. Wash-resistance index was calculated according to WHO guidelines [22] as indicated below:

$${\text{Wash}}\;{\text{resistance}}\;{\text{index}} = 100 \times {\text{n}}\sqrt {\left( {{\text{t}}{\mathbf{n}}/{\text{t}}{\mathbf{0}}} \right)} \;\left( {{\text{free}}\;{\text{migration}}\;{\text{stage}}\;{\text{behaviour}}} \right)$$

where t**n** = total active ingredient content after n washing cycles, t**0** = total active ingredient content before washing, n = number of washes.

### Data analysis

Proportional outcomes (blood-feeding, exiting and mortality) related to each experimental hut treatment (unwashed and washed 20 times) were assessed using binomial generalized linear mixed models (GLMMs) with a logit link function, fitted using the ‘lme4’ package for R (version 3.5.3). A separate model was fitted for each outcome. In addition to the fixed effect of each treatment, each model included random effects to account for the following sources of variation: between the huts; between the sleepers; between the weeks of the trial; and finally, an observation-level random effect to account for variation not explained by the other terms in the model (over dispersion).

### Ethical considerations

This study received ethical approval from the Ministry of Health in Benin and from the Ethics Review Committee of the London School of Hygiene & Tropical Medicine. Informed consent was obtained from each human volunteer sleeper who slept in the huts to attract mosquitoes prior to their participation. Sleepers were also offered chemoprophylaxis. Through the course of the study, they were examined regularly for signs of fever by a stand-by nurse; any sleepers testing positive for malaria were withdrawn from the study and treated properly.

## Results

### Insecticide resistance in malaria vectors in Cove

Mortality with permethrin and alpha-cypermethrin treated papers was 100% with the laboratory-maintained pyrethroid-susceptible *An. gambiae* Kisumu strain. With wild pyrethroid resistant *An. gambiae *s.l. from Cove, mortality rates were < 50% with all three pyrethroid insecticides tested (Table 1) thus confirming the high levels of pyrethroid resistance in this vector population. Mortality however increased from 19.2 to 52.5% with permethrin, 41.7% to 69.7% with deltamethrin and 0% to 82.4% with alphacypermethrin after pre-exposure of the Cove strain to PBO synergist (Table 1). This result demonstrated that mixed function oxidases are overexpressed in the wild Cove vector population and their effect can be effectively inhibited by the PBO synergist.

**Table 1:** Mortality of pyrethroid resistant *Anopheles gambiae* s.l. from Cove in WHO cylinder bioassays with and without pre-exposure to PBO

	N exposed	N dead	% Dead
Control	101	0	0.0
PBO 4% only	103	3	2.9
Permethrin 0.75%	104	20	19.2
Deltamethrin 0.05%	91	38	41.7
Alpha-cypermethrin 0.05%	102	0	0.0
PBO 4% then permethrin 0.75%	99	52	52.5
PBO 4% then deltamethrin 0.05%	99	69	69.7
PBO 4% then alpha-cypermethrin 0.05%	102	84	82.4

### Experimental hut trial results

#### Mosquito entry and exiting rates in experimental huts

A total of 6711 pyrethroid resistant female *An. gambiae *s.l. were collected during the experimental hut trial. The entry and exiting rates of wild pyrethroid resistant *An. gambiae *s.l. from the experimental huts with the different ITN types tested in the trial are presented in Table 2. Compared to the control, Olyset Net did not deter mosquitoes from entering the experimental huts (0% when unwashed and after 20 washes). Mosquito deterrence was significantly higher with PermaNet 3.0 compared to Olyset Plus both unwashed (49% vs. 28%, P < 0.001) and after 20 washes (36% vs. 0%, P < 0.001). Nevertheless, early exiting of mosquitoes from the experimental huts into the veranda trap did not differ significantly between both pyrethroid-PBO net types both when unwashed (65% with PermaNet 3.0 vs 68% with Olyset Plus, P = 0.232) and after 20 washes (60% with PermaNet 3.0 vs. 56% with Olyset Plus, P = 0.053).

**Table 2:** Entry and exiting rates of wild pyrethroid resistant *Anopheles gambiae* s.l. in experimental huts in Cove, Benin

Net type	Control	Olyset Net		PermaNet 3.0		Olyset Plus	
Number of washes	0	0	20	0	20	0	20
N collected	962	1135	1546	489	616	688	1275
N females/night	23^a^	27^ab^	37^b^	12^c^	15^d^	16^d^	30^ab^
% deterrence	–	0*	0*	49	36	28	0
N exiting	429	639	669	319	370	468	712
% exiting	45^a^	56^b^	43^a^	65^ cd^	60^ce^	68^d^	56^be^
95% conf. limits	41.5–47.7	53.4–59.2	40.8–45.7	61.0–69.5	56.2–63.9	64.4–71.4	53.1–58.6

Values along a row bearing the same letter label are not significantly different (P > 0.05)

^*^Value set to zero as more mosquitoes caught in Olyset Net huts than control huts

#### Mortality of wild pyrethroid-resistant *Anopheles gambiae* s.l. in experimental huts

**Fig. 1:**
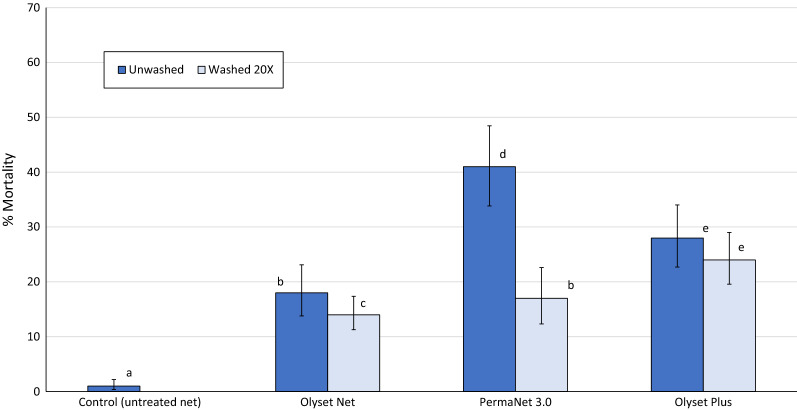
Mortality of wild pyrethroid-resistant *Anopheles gambiae* s.l. in experimental huts in Cove, Benin evaluating different net types. Vertical lines indicate 95% confidence interval estimates whilst bars with the same letter label are not significantly different (P > 0.05)

Mortality rates of wild pyrethroid resistant mosquitoes that entered the experimental huts are presented in Fig. 1 with further details provided in Table 3. The lowest mortality was achieved with Olyset Net (18% before washing and 12% after 20 washes). Percentage mortality with unwashed pyrethroid-PBO nets was highest with PermaNet 3.0 (41%) but this declined significantly after 20 washes (17%, P < 0.001). Mortality with unwashed Olyset Plus was 28% and while this value was significantly lower than the mortality shown by unwashed PermaNet 3.0 (P < 0.001), it did not decrease significantly after 20 washes (28% vs. 24%, P = 0.433) whereas for PermaNet 3.0 it declined. Hence, Olyset Plus induced higher mortality rates than PermaNet 3.0 after 20 washes (24% vs 17%, P < 0.001). With respect to the pyrethroid-only ITN, both pyrethroid-PBO nets induced significantly higher mortality rates than Olyset Net with nets washed 20 times (P < 0.001) though the difference was higher with Olyset Plus (24% vs. 12%), than with PermaNet 3.0 (17% vs. 12%). After 20 washes, mortality with PermaNet 3.0 declined to the same level as the unwashed Olyset Net, (17% vs. 18%, P = 0.061) but remained significantly higher with Olyset Plus (24% vs. 17%, P = 0.036).

**Table 3:** Mortality of wild pyrethroid resistant *Anopheles gambiae* s.l. in experimental huts in Cove, Benin

Net type	Control	Olyset Net		PermaNet 3.0		Olyset Plus	
Number of washes	0	0	20	0	20	0	20
Total collected	962	1135	1546	489	616	688	1275
Total dead	12	209	284	198	103	190	297
Mortality (%)	1^a^	18^b^	12^c^	41^d^	17^b^	28^e^	24^e^
95% conf. limits	0.6–2.0	16.2–20.1	10.3–13.5	36.1–44.8	13.8–19.8	24.4–31.1	21.0–25.6
Corrected for control (%)	–	17	11	41	17	27	22

Values along a row bearing the same letter label are not significantly different (P > 0.05)

#### Mosquito blood-feeding rates in experimental huts

**Fig. 2:**
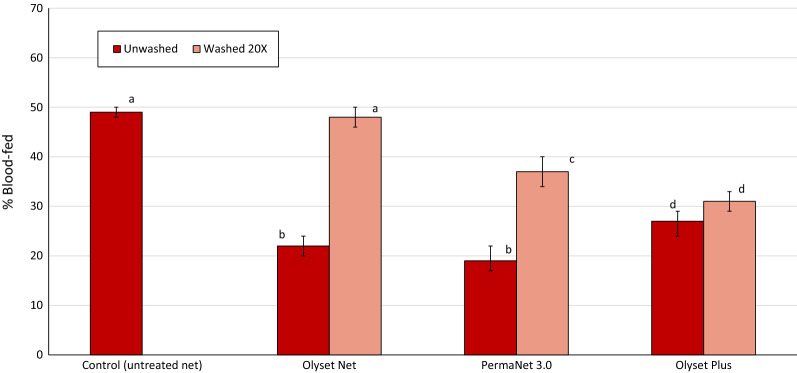
Blood-feeding rates of wild pyrethroid-resistant *Anopheles gambiae* s.l. in experimental huts in Cove, Benin evaluating different net types. Vertical lines indicate 95% confidence interval estimates whilst bars with the same letter label are not significantly different (P > 0.05)

The blood-feeding rates of wild pyrethroid-resistant *An. gambiae s.l* that entered the experimental huts are presented in Fig. 2 with further details on mosquito feeding provided in Table 4. The percentage blood-feeding was lower in huts with the unwashed pyrethroid-PBO ITNs and was lowest of all with unwashed Olyset Plus as compared to unwashed PermaNet 3.0 (8% vs 19%, P < 0.001). For all net types, the data showed an overall increase in blood feeding with washed nets compared to unwashed nets and a decrease in blood-feeding inhibition relative to the untreated net. Percentage blood-feeding when washed 20 times did not differ significantly between the pyrethroid-PBO types (38% with PermaNet 3.0 vs. 35% with Olyset Plus, P = 0.708). The proportions of mosquitoes collected resting in the nets were lowest with the unwashed pyrethroid-PBO nets (2–6%), which is consistent with the higher toxicity observed with this type of net. Personal protection with both types of pyrethroid-PBO ITNs were > 80% when unwashed but this declined after 20 washes to 53% with PermaNet 3.0 and 11% with Olyset Plus.

**Table 4:** Blood-feeding rates of wild pyrethroid resistant *Anopheles gambiae* s.l. in experimental huts in Cove, Benin

Net type	Control	Olyset Net		PermaNet 3.0		Olyset Plus	
Number of washes	0	0	20	0	20	0	20
Total collected	962	1135	1546	489	616	688	1275
Total blood fed	496	324	807	95	231	56	442
% Blood fed	52^a^	29^b^	52^a^	19^c^	38^d^	8^e^	35^d^
95% conf intervals	48.4–54.7	25.9–31.2	49.7–54.7	15.9–22.9	33.7–41.3	6.1–10.2	32.1–37.3
% Blood-feeding inhibition	–	44	0	63	27	85	33
inside net	294	184	542	28	130	17	241
inside net (%)	31^a^	16^bc^	35^a^	6^d^	21^b^	2^e^	19^c^
Personal protection (%)	–	35	0*	81	53	89	11

Values along a row bearing the same letter label are not significantly different (P > 0.05)

^*^Value set to zero as more blood fed mosquitoes were caught in washed Olyset Net huts than control hut

#### Non-inferiority assessment

According to recent provisional WHO guidelines [19], for a candidate pyrethroid-PBO ITN product to be included in this new WHO intervention class without the need for epidemiological evidence, it must demonstrate non-inferiority to the first in class product which has already demonstrated public health value (Olyset Plus) and superiority to a pyrethroid-only LLIN in experimental hut trials [19]. Briefly, the candidate pyrethroid-PBO product is deemed non-inferior if: (1) The lower 95% confidence interval estimate of the odds ratio describing the difference in mosquito mortality between the candidate and active comparator product is greater than 0.7. and (2) The upper 95% confidence interval estimate of the odds ratio describing the difference in mosquito blood-feeding between the candidate and active comparator product is less than 1.43. Following the WHO guidelines, both unwashed and washed data of each product were analysed together to generate single estimates of efficacy representative of the overall performance over the lifetime of the product in the field.

Each primary endpoint for non-inferiority (mortality and blood-feeding rate for unwashed and washed nets combined), was assessed using binomial generalized linear mixed models (GLMMs) with a logit link function fitted using the ‘lme4’ package of R version 3.5.3 for Windows as described earlier. Results from the non-inferiority assessment of PermaNet 3.0 to Olyset Plus are presented in Table 5 below. The odds ratio for the difference in mosquito mortality between PermaNet 3.0 and Olyset Plus was 1.528 (95% confidence interval: 1.021–2.289) while the odds ratio for the difference in mosquito blood feeding was 1.192 (95% confidence interval: 0.772–1.841). Following the WHO criteria described above, PermaNet 3.0 was non-inferior to Olyset Plus in terms of its ability to kill wild pyrethroid- resistant *An. gambiae* s.l. in the experimental hut trial in Cove Benin. In contrast PermaNet 3.0 was not non-inferior to Olyset Plus in terms of proportions of mosquitoes that blood-fed and, therefore, fails to demonstrate non-inferiority for blood feeding inhibition in this trial. The results also showed superiority of both pyrethroid-PBO net types to Olyset Net both in terms of mosquito mortality (25–27% vs. 15%, P < 0.001) and reducing blood-feeding (25%-30% vs. 42%, P < 0.001).

**Table 5:** Results from the non-inferiority assessment of PermaNet 3.0 to Olyset Plus against wild pyrethroid-resistant *Anopheles gambiae* s.l. in experimental huts in Cove, Benin

	Control Net	Olyset Net	Olyset Plus	PermaNet 3.0
Total collected	962	2681	1963	1105
Mortality				
Total dead	12	393	488	301
Mortality (%)	1	15	25	27
Odds ratio	–	–	–	1.528
Std. err (on log odds scale)	–	–	–	0.206
95% conf. interval	–	–	–	1.021–2.289
WHO non-inferiority margin	–	–	–	Lower 95%CI > 0.7
Conclusion	–	–	–	Non-inferior
Blood-feeding rate				
Total blood-fed	496	1131	498	326
Blood-feeding (%)	52	42	25	30
Blood-feeding inhibition	–	19	52	42
Odds ratio	–	–	–	1.192
Std. err (on log odds scale)	–	–	–	0.222
95% conf. interval	–	–	–	0.772–1.841
WHO non-inferiority margin	–	–	–	Upper 95%CI < 1.43
Conclusion	–	–	–	Not non-inferior

Combined data for washed and unwashed nets of each net type

### Supplementary laboratory bioassays results

#### Cone bioassay results

**Fig. 3:**
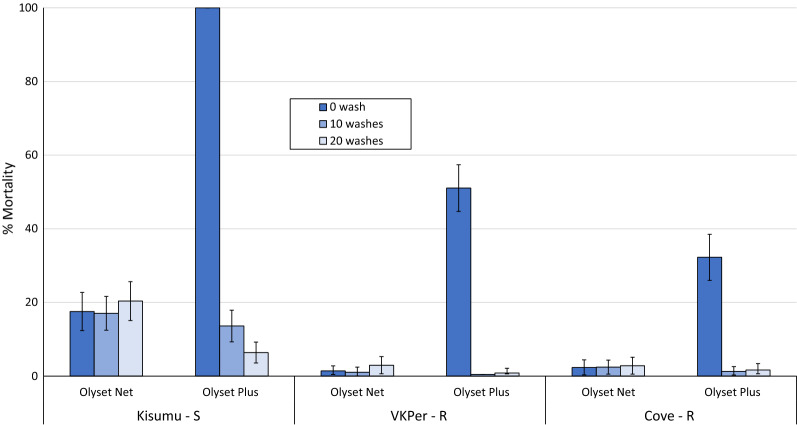
Mortality (%) of susceptible and resistant strains of *An. gambiae* s.l. in cone bioassays with Olyset Plus and Olyset Net. S = susceptible, R = resistant

The 3-min cone bioassay mortality results for all 3 mosquito strains and wash points tested are presented in Fig. 3. Unwashed Olyset Net induced very low mortality rates against the susceptible Kisumu strain (17–20%) and even lower mortality rates against the pyrethroid resistant strains (< 5%). Cone bioassay mortality rates with unwashed Olyset Plus were higher across all 3 strains compared to Olyset Net though mortality decreased as the strain tested became more pyrethroid-resistant. Cone bioassay mortality however dropped significantly with washed net samples. Mortality with the untreated net samples did not exceed 5% with any strain tested.

#### Tunnel test results

**Fig. 4:**
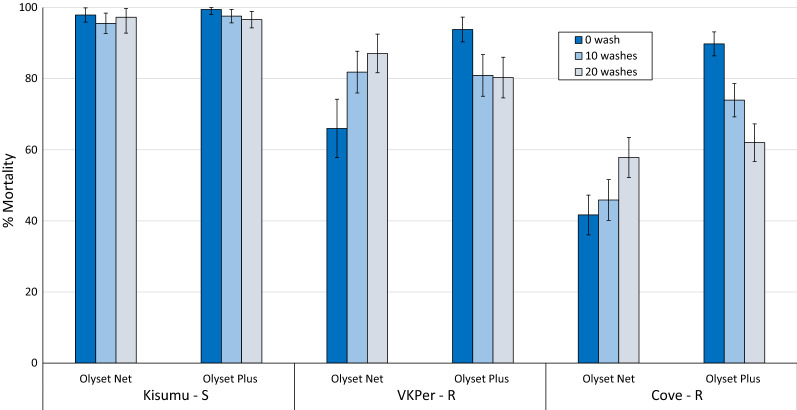
Tunnel test mortality (%) of susceptible and resistant strains of *Anopheles gambiae* s.l. exposed to Olyset Plus vs. Olyset Net

**Fig. 5:**
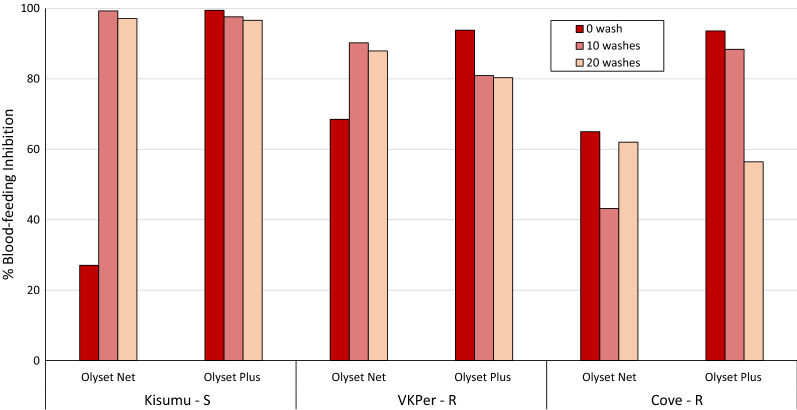
Blood-feeding inhibition (%) of susceptible and resistant strains of *An. gambiae s.l.* in tunnel tests with Olyset Plus and Olyset Net. Blood-feeding inhibition was calculated relative to the control tunnel. S = susceptible, R = Resistant

The results from the tunnel tests comparing Olyset Plus and Olyset Net unwashed and after 10 and 20 washes against all three strains are presented in Figs. 4 and 5 for mortality and blood-feeding inhibition respectively. Mortality rates were generally higher in the tunnels tests compared to the cone bioassays and decreased as the strain become more pyrethroid-resistant (Fig. 4). Mortality rates with the Kisumu strain were very high with both Olyset Net and Olyset Plus (> 95%). With the VKPer strain, mortality remained > 80% after 20 washes with both ITN types. With the Cove strain, mortality was significantly higher with Olyset Plus compared to Olyset Net at 0 and 10 washes but about the same after 20 washes. With unwashed nets, blood-feeding inhibition in the tunnel tests was consistently higher with Olyset Plus (> 90%) compared to Olyset Net (27–75%) for all three strains tested (Fig. 5). After 10 and 20 washes, blood-feeding inhibition of the Kisumu and VKPer strain remained > 80% with Olyset Plus and Olyset Net. With Cove strain, blood-feeding inhibition was also higher with Olyset Plus at 0 and 10 washes but declined to about the same level as Olyset Net after 20 washes (56%). Mortality in the untreated control tunnel was < 10% with all three strains.

### Chemical analysis results

For PermaNet 3.0, PBO was only recorded from the roof panel of the nets; for Olyset Plus, PBO was recorded on all 5 panels (Table 6). Compared to the pyrethroid component, much less PBO was retained in both types of pyrethroid-PBO ITNs after 20 washes (58.7% vs. 25% with Olyset Plus and 80.9% vs. 68.8% with PermaNet 3.0, P < 0.05). The decrease in PBO content after washing was more evident in Olyset Plus than in PermaNet 3.0 netting, hence the wash retention index of PBO was higher with PermaNet 3.0 compared to Olyset Plus (98.1% vs. 93.3%).

**Table 6:** Chemical analysis of net samples after experimental hut trial at Cove, Benin

LN type	Active ingredient	AI content (g/kg)		Retention, %	Wash retention index (%)
		Unwashed	Washed 20×		
Olyset Net	Permethrin	12.0	12.0	100.0	100.0
Olyset Plus	Permethrin	15.5	9.1	58.7	97.4
	PBO	8.0	2.0	25.0	93.3
PermaNet 3.0	Deltamethrin (roof of net)	4.2	3.4	80.9	98.9
	PBO (roof of net)	16.0	11.0	68.8	98.1

## Discussion

Following the interim endorsement of pyrethroid-PBO nets by the WHO [23], malaria vector control programmes are faced with additional choice of different brands of prequalified pyrethroid-PBO nets [7]. Considering the wide variations in design of the available brands of these nets, studies generating the required assurance of comparative performance to Olyset Plus (the first in class pyrethroid-PBO net to demonstrate empirical evidence of entomological and epidemiological impact) are necessary. This study compared the efficacy and assessed the non-inferiority of PermaNet 3.0 to Olyset Plus in experimental huts against pyrethroid resistant malaria vectors in a highly endemic country of West Africa, following WHO guidelines [19, 22].

The WHO susceptibility bioassays confirmed the high levels of pyrethroid resistance in the vector population at the experimental hut site during the trial, corroborating previous findings [20]. The increased mortality achieved in bioassays with pre-exposure to PBO showed that pyrethroid-resistance was indeed at least partly mediated by increased mono-oxygenase activity. The experimental hut trial demonstrated improved levels of mortality and blood-feeding inhibition with both pyrethroid-PBO ITN types compared to a standard pyrethroid-only LLIN against a vector population that was very resistant to pyrethroids; this was supported by results from the laboratory assays which compared Olyset Plus to Olyset Net against pyrethroid-resistant mosquito strains. These observations are partly attributable to the synergistic effect of the PBO on pyrethroid resistance and are consistent with other experimental hut trials across Africa and epidemiological trials performed in Tanzania [8] and Uganda [18].

Twenty washes in experimental hut studies are indicated by WHO as a proxy for the ability of an ITN to withstand multiple washes under operational use over a 3-year life span [19, 22]. With unwashed nets, the levels of improved mortality relative to the standard pyrethroid-only net were higher with PermaNet 3.0 than with Olyset Plus. However, unlike with Olyset Plus, this effect was lost with PermaNet 3.0 after twenty washes; PermaNet 3.0 killed significantly lower proportions of mosquitoes than Olyset Plus and same proportions as an unwashed pyrethroid-only ITN. Though the RCT in Uganda was not powered to assess differences between the pyrethroid-PBO ITN brands tested (PermaNet 3.0 vs. Olyset Plus) [18], the results from our trial appear consistent with some of the differences in epidemiological effect observed between both brands: (1) The high experimental hut mortality with the unwashed PermaNet 3.0 supports the higher initial protective effect observed with the net in the Ugandan trial at the 6 months epidemiological survey which was not seen with Olyset Plus. (2) The higher rate of decline in experimental hut mortality after washing with PermaNet 3.0 compared to Olyset Plus is consistent with the shorter-lived epidemiological effect in the Ugandan trial with PermaNet 3.0 (up to 12 months) compared to Olyset Plus which remained more protective than the pyrethroid-only net at 18 months. However, care should be taken to not over-interpret the comparisons between results from our hut trial and Ugandan RCT considering the different geographical settings and the lack of sufficient power to differentiate between the epidemiological impact of both pyrethroid-PBO net type in the Ugandan trial.

The difference in hut performance between both pyrethroid-PBO ITNs can be attributed to differences in the retention and movement of PBO across the polymer fibre in Olyset Plus compared to PermaNet 3.0 and/or differences in design and specification. Retention of bioefficacy of pyrethroid-PBO nets is a fine balance between migration and replenishment of PBO from the core to the surface of fibres and the maintenance of an internal reservoir sufficient to last the lifespan of the LLIN, which is typically set at 3 years of use [24]. The chemical analysis results showed a faster release of PBO in the Olyset Plus netting after 20 washes compared to PermaNet 3.0 though it is unclear whether this may have increased the bioavailability of the PBO on the surface of Olyset Plus after washing. Whether sufficient PBO would remain within the fibres of both pyrethroid-PBO nets after 3 years of household use is presently unknown and is the subject of ongoing WHO durability trials of Olyset Plus and PermaNet 3.0 which are not yet completed [18, 25]. Another factor which may contribute to the discrepancies in efficacy of the two pyrethroid-PBO ITNs are differences in design and specification: Olyset Plus is treated with the pyrethroid permethrin while PermaNet 3.0 is treated with deltamethrin, and Olyset Plus contains PBO on every panel whereas in PermaNet 3.0, PBO is available only on the top panel of the net. It is not clear whether the restricted application of PBO to the roof of the net would affect bioefficacy. This would require comparative trials of the PermaNet 3.0 with pyrethroid-PBO restricted to the upper panel and pyrethroid to side panels versus an ITN with all 5 panels treated with pyrethroid-PBO. Behavioural studies of mosquitoes around nets indicate that mosquitoes may first make multiple contacts with the roof panel in response to odour plumes [26]; however, experimental hut trials comparing restricted versus full PBO coverage on nets are too few to be definitive on the question of efficacy.

Despite the differences in performance observed between both pyrethroid-PBO net types with regards to their impact after 20 standardized washes, the non-inferiority analysis performed in accordance with recent WHO guidelines [19] showed that PermaNet 3.0 was non-inferior to Olyset Plus in terms of mosquito mortality but not with blood-feeding inhibition. The higher blood-feeding inhibition observed with Olyset Plus could be due to the high excito-repellency of permethrin in Olyset Plus compared to deltamethrin in PermaNet 3.0 [27]. Alternatively, the study may not have had sufficient power to demonstrate non-inferiority of PermaNet 3.0 to Olyset Plus for both endpoints; further studies are on-going to help guide power calculations for ITN non-inferiority studies. The non-inferiority margin used for the analysis was defined by WHO as an odds ratio of 0.7 in mosquito mortality and feeding between a candidate net and the first in class net considered acceptable for both products to be in the same policy class. According to these guidelines, if non-inferiority is demonstrated in two independent experimental hut trials in different geographical locations representative of where the products will be deployed, then PermaNet 3.0 will be placed in the same WHO vector control product class as Olyset Plus [19]. It is however not clear whether non-inferiority must be demonstrated for both endpoints (mortality and blood-feeding) for a second-in class product to become part of an intervention class. While the guidelines were developed more like a compromise between the risk of accepting an inferior product and the feasibility of conducting epidemiological trials, the findings from our trial show that non-inferiority experimental hut trials are complex, and results must be interpreted with care. Comparative performance between products may also depend on other location-specific factors, such as the intensity of insecticide resistance and behaviour of the target vector population which should be taken into consideration when choosing between products of the same class.

While the present hut trial in Benin and earlier hut trials in Benin, Tanzania, Cameroon, Burkina Faso, Côte d’Ivoire and Vietnam where the vectors were also resistant have shown some additional effect of pyrethroid-PBO nets over a standard pyrethroid net [12, 13, 15–17], the margin appears to vary depending on the level of pyrethroid-resistance encountered [28]. The absolute increase in hut trial mortality with Olyset Plus compared to Olyset Net in the present study (24–28% vs. 12–18%) conducted in an area of intense pyrethroid-resistance [20] is lower than what has been previously reported with Olyset Plus in another area in Northern Benin where pyrethroid resistance was less prevalent (67–81% vs. 36–42%) [16]. Compared to East Africa, West Africa has shown historically higher intensity of pyrethroid resistance in malaria vectors [29] mediated by complex and multiple insecticide resistance mechanisms which may not be effectively tackled by the synergistic effects of PBO in pyrethroid-PBO ITNs [4, 23]. It is therefore not clear whether a diminishment in experimental hut mortality with pyrethroid-PBO nets due to increasing intensity of pyrethroid-resistance would translate to a reduced epidemiological effect of pyrethroid-PBO ITNs in West Africa compared to East Africa which has been the site of the only epidemiological trials so far.

## Conclusion

Olyset Plus outperformed PermaNet 3.0 in terms of its ability to induce improved levels of mosquito mortality compared to a standard pyrethroid LLIN after multiple standardized washes. Nevertheless, a non-inferiority analysis of both ITN types following recent WHO guidelines showed that they were comparable in their ability to kill mosquitoes. Compared to the situation existing in Benin several years ago, both pyrethroid-PBO ITNs showed less impact against the mosquito vector due to increased levels of resistance. West Africa constitutes a different environment and ecology from East Africa, with historically higher intensity of pyrethroid resistance in malaria vectors. It is not clear whether either of these nets would have the same epidemiological impact against malaria demonstrated with pyrethroid-PBO nets in trials in East Africa. A cluster randomized trial of pyrethroid-PBO nets with epidemiological outcome indicators is urgently required in West Africa to establish its effectiveness against malaria.

## Data Availability

The datasets used and/or analysed during the current study are available from the corresponding author on reasonable request.

## Abbreviations

ITN: Insecticide treated nets
LLIN: Long-lasting insecticidal nets
PBO: Piperonyl butoxide
WHO: World Health Organization
PQ: Prequalification team
WHOPES: WHO Pesticide Evaluation Scheme
CREC: Centre de Recherche Entomologique de Cotonou
LSHTM: London School of Hygiene & Tropical Medicine
HPLC: High Performance Liquid Chromatography
Kdr: Knock down resistance
